# OSCAR is an online ML-powered tool for organoid cell counting using bright-field images

**DOI:** 10.1016/j.crmeth.2025.101251

**Published:** 2025-12-02

**Authors:** Stephanie E.A. Burnell, Lorenzo Capitani, Chloe A. Harris, Luned M. Badder, Alan L. Parker, Kasope Wolffs, Yuan Chen, Andrew J. Godkin, Awen M. Gallimore

**Affiliations:** 1 Division of Infection and Immunity, School of Medicine, Cardiff University, Heath Park, Cardiff CF14 4XN, UK; 2 Division of Cancer and Genetics, School of Medicine, Cardiff University, Heath Park, Cardiff CF14 4XN, UK; 3 Wales Cancer Biobank, UHW Main Building, Heath Park, Cardiff CF14 4XN, UK; 4 Department of Gastroenterology and Hepatology, University Hospital of Wales, Heath Park, Cardiff CF14 4XN, UK

**Keywords:** organoid, *in vitro*, Mask R-CNN, modeling, quantification, cell number quantification, co-cultures

## Abstract

Numerous software tools have been published to aid organoid quantification. These tools generate estimates of total organoid number and morphological characteristics in images. However, there remains a need to estimate the number of organoid cells in a well for use in organoid-based experiments (e.g., co-cultures). We present OSCAR (organoid segmentation and cell number approximation using regression), a workflow for estimating organoid cell numbers from bright-field images. Step one is a Mask-R-CNN-based convolutional neural network for identifying organoids in bright-field images and estimating the area of each organoid. Step two is an empirical multiple linear regression model relating the number of cells in an organoid to its area. OSCAR’s estimate of the total number of cells in a well was within ±16% of the real number of organoid cells. OSCAR is an online tool capable of generating this key metric and will contribute to the increased reliability of organoid-based assays.

## Highlights

• A Mask R-CNN model for precise organoid segmentation and cell counting
• Accurately estimates organoid cell numbers from bright-field images
• Provides an additional tool to enhance reliability of organoid-based assays

## Motivation

Organoids are increasingly being used as *in vitro* models; however, methods for obtaining estimates of organoid cell numbers for use in experiments are limited. Obtaining such a metric could aid in streamlining organoid experiments while improving their reproducibility. Addressing this bottleneck, this study combines computer vision and mathematical modeling approaches, providing a method for estimating the number of organoid cells in a well from a bright-field microscopy image alone and showing its use in a co-culture assay setting.

## Graphical abstract

**Figure:**
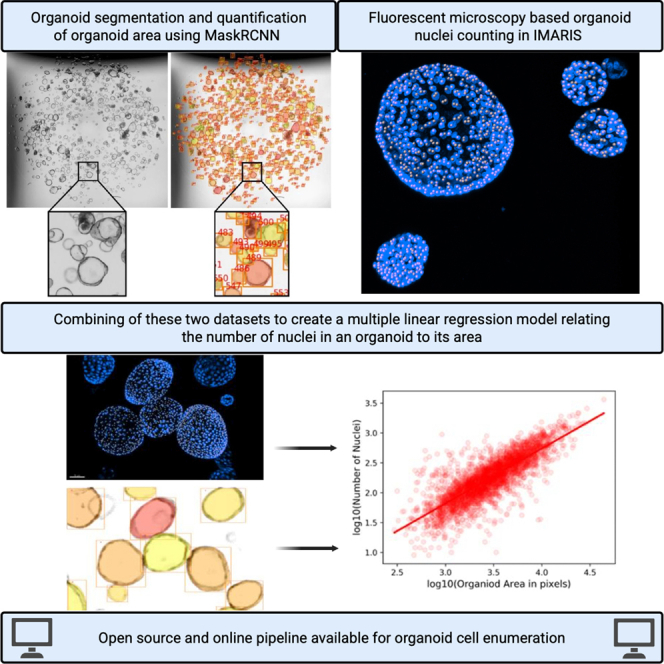

## Teaser

Burnell et al. develop a computer-based method to count the number of cells in a well of organoids from a regular microscope image. They compare its use to standard counting techniques and test it in experiments where organoids are cultured with immune cells.

## Introduction

The importance and utility of *in vitro* systems for the characterization of immune function (and beyond) cannot be understated. Classic culture systems for *in vitro* experiments have typically utilized immortalized or primary cells either in suspension or adherent to 2D surfaces. Albeit useful, these systems are greatly limited in how faithfully they recapitulate the complex *in vivo* three-dimensional physiological environments. As a result, they quite commonly lead to findings that are not predictive of *in vivo* physiology.^1^

To combat this, much research in recent years has been devoted to 3D cultures, including spheroids and organoids. Specifically, organoids, also known as mini-organs, are multicellular structures derived from primary cells or stem cells, and in many ways, they resemble the native organ they are derived from, across cellular, structural, and functional levels.^2^ Given this, organoids are rapidly becoming the *in vitro* system of choice for a wide range of applications, from drug testing to the study of organ development.

In this context, the growth of organoids is not a simple task and has required extensive years of research to narrow down ideal growth conditions, which has, in part, been informed by the study of their morphology (e.g., shape or size). However, in our experience with colorectal organoids, they are small in size (average of 50–150 μm) and are grown within a biological hydrogel substrate at quite confluent levels. Their enumeration is typically performed by dissociating the organoids into single cells and counting using techniques such as flow cytometry, cell counters, or a hemocytometer, which can be a time-consuming task that is additionally problematic when the organoids are required whole (i.e., not dissociated) for experimentation.

To overcome the above, recent efforts have attempted to automate organoid quantification to overcome human-derived errors in a standardized fashion. However, existing approaches have limitations, including (1) being limited in the morphology of organoids that they can recognize, (2) making it difficult to identify organoids in output files, (3) requiring stitching of multiple image sections in secondary software for the enumeration of whole wells, and finally (4) providing the total number of organoids in the well as their primary output metric. Recent publications have overcome some of these limitations. For example, numerous organoid segmentation methods now display robustness in recognizing a range of organoids,^3^^,^^4^ and the recent SiQ-3D enables single-cell tracking of organoids but requires fluorescent marking.^5^

Importantly, amid a “reproducibility crisis,”^6^ the standardization of *in vitro* assays is of the utmost importance. Organoids have become a model of choice for carrying out immune co-cultures, in which immune cells are cultured with organoids for a wide range of applications, such as cytotoxicity assays. In this context, knowing the number of cells in a well is a crucial parameter in establishing the effector-to-target (E:T) ratio used in an experiment, a metric that can substantially impact readouts. Existing computational approaches are inadequate to address this issue, as, although they can enumerate the number of organoids in a well, given how widely organoids range in size, it is highly likely that two wells having the same number of organoids possess a completely different number of individual cells. Equally, existing computational single-cell quantification methods require fluorescent marking, which can be inadequate depending on the experimental setup.^5^ We sought to develop a dye-free organoid cell number estimation method. To this end, we present “organoid segmentation and cell number approximation using regression” (OSCAR), a tool inspired by the shortcomings of previously published organoid cell enumeration software. OSCAR is a Python-based tool that employs an initial instance-segmentation-based approach to recognize organoids from bright-field microscopy images. OSCAR contains a secondary step in which an empirically generated multiple linear regression model utilizes the area of each detected organoid instance to generate an estimate of the number of cells in said organoid. OSCAR also includes an optional step that streamlines the process for users requiring image stitching, significantly cutting down the manual “hands-on” time necessary for large datasets from hours to minutes. Beyond OSCAR itself, we further provide a methods protocol for users who wish to develop empirical models for their own samples and provide users with the option to input their model into the OSCAR pipeline. Our software is not without limitations, providing an estimate of the number of organoid cells in a well, deviating from the real cell number by an average of 16% (95% confidence interval [CI] [11%–20%]) as opposed to an absolute cell count. Nonetheless, OSCAR takes an important step forward in standardizing organoid-based co-culture assays: by combining these distinct processes described, OSCAR can be accessed by all and provides an estimate of the total number of cells within a well of organoids simply from a bright-field microscopy image, an extremely useful metric that, to our knowledge, was previously impossible to generate without dissociating organoids to single-cell suspensions or employing fluorescent-dye-based methods.

## Results

### Organoid segmentation using Mask R-CNN

**Figure 1:**
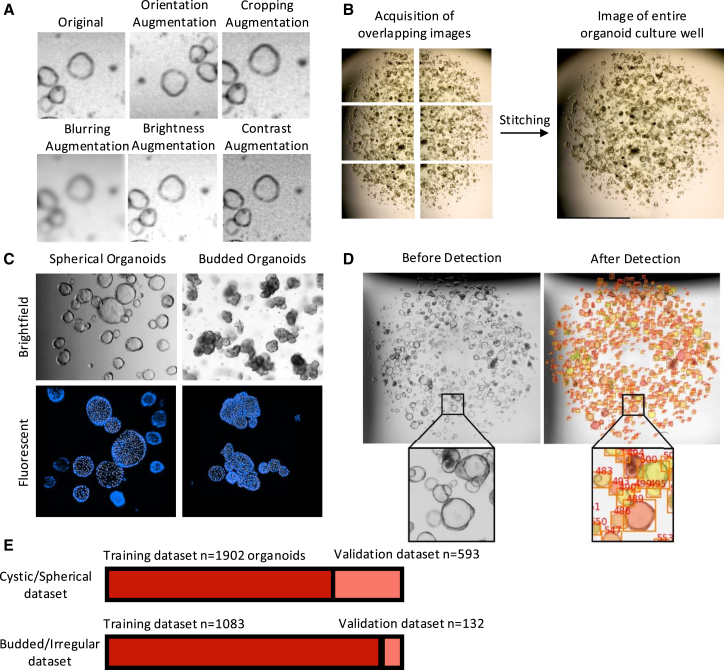
Development of the organoid detection model (A) Image augmentation procedures. Original images were subject to image augmentation procedures to ensure the model could recognize the organoids in a variety of conditions. (B) Creation of an image containing the entire well of organoids. Overlapping images were obtained using an EVOS bright-field microscope; these were then stitched together with the OpenCV2 Stitcher Class in Python in our analysis pipeline. (C) Two distinct models were developed, one capable of detecting cystic organoids and one for the detection of budded organoids. (D) Detection and identification of organoids in a bright-field image. On the left is a bright-field microscopy image of an organoid culture well. On the right is the same image following analysis with the Mask R-CNN model, showing organoids detected and given a unique ID. Organoids are colored to allow for ease of identification and numbers to allow for downstream analysis of individual organoids in the image. See also Figures S1 and S2.

To maximize the size of the input data and to promote a robust model capable of recognizing objects of interest under a range of conditions and to equally limit overfitting, we employed data augmentation. These context-dependent transformations to which data are exposed increase the dataset size and the breadth of input image parameters. This is a standard procedure in the computer vision field when used in a context-dependent fashion, in this instance, bright-field microscopy images of organoids (Figure 1A). To enable the acquisition of entire organoid wells, we included a stitching step in our pipeline (Figure 1B).

From the numerous model architectures available, we decided to employ the Mask R-CNN model architecture, given its high accuracy.^7^^,^^8^ To simplify model development, we developed two distinct models, one capable of detecting cystic organoids and one for the detection of budded organoids, resulting in an image highlighting the detected organoids within the whole well (Figures 1C and 1D). The size of our input datasets for each model is shown in Figure 1E, with each image being 512 × 512 pixels. Importantly, to ensure that each organoid in the CSV file can be mapped to an organoid in the output image, each organoid is given a unique numeric ID, which is displayed in the image and included in the CSV file. This enables downstream analysis of individual organoids in the original image. Different organoids are also given random colors to enable the distinction of overlapping/adjacent organoids in crowded images.

A key hurdle in the development of this pipeline was ensuring that our model could be rapidly used on large images of any size. In our hands, an imaged organoid well could be anywhere between 2,000 and 5,000 pixels in either dimension. Given their size, repeatedly loading such large images posed computational memory constraints.

To overcome this challenge, inspiration was taken from a recent publication that employed a Mask R-CNN model, for instance, segmentation on images taken by satellites. The authors apply the model to very large images by splitting the image into quadrants and then imposing the instance segmentations of each quadrant onto the original image, a process we will refer to as “mosaicking.”^9^ This is further described in the Figure S1. Once all tiles have been analyzed by the model and each organoid occurs across all tiles just once (Figures S2A–S2C), the data from all tiles are merged into a single image with dimensions equal to the original input image.

The performance of our model was measured by calculating its mean average precision (mAP), a routinely used metric to evaluate the performance of object detection models. mAP considers the precision (i.e., the number of true positives divided by the number of total positives) and the recall or sensitivity (i.e., the number of true positives divided by the sum of true positives and false negatives). A perfect model capable of detecting all objects in an image would have an mAP of 1. The model developed to identify cystic organoids (circular) showed a very robust mAP of 0.855, while the model developed to recognize budded (irregularly shaped) organoids displayed a similarly robust mAP of 0.81. Furthermore, to establish the best confidence threshold to use for each model, an ROC curve was plotted (Figures S2D and S2E). The ROC curve for the cystic model showed that a threshold of 0.795 showed the best performance, while that for the budded model showed that a threshold of 0.836 was best. These values were then taken forward and used as the default confidence thresholds for downstream applications.

### Empirical model development to relate the number of organoid cells in an organoid to its estimated area

**Figure 2:**
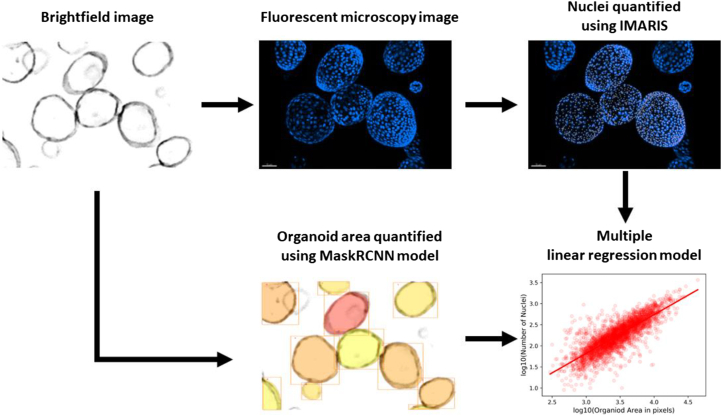
Detection and analysis of organoid cell numbers by the OSCAR pipeline using both bright-field and fluorescent microscopy From the same well of organoids, a bright-field image is taken to be quantified with the Mask R-CNN model, and in parallel, the organoids are stained with Hoechst to detect the nuclei present. These are quantified using the spots selection tool on IMARIS. This information is then fed into a multiple linear regression model to predict the number of nuclei in an organoid based on its size in future experiments. See also Figures S1 and S2.

The second main feature of OSCAR is an empirical multiple linear regression model that relates the area of an organoid to the number of nuclei in it. We manually matched the number of nuclei in fluorescently labeled organoid images to their respective bright-field images, alongside the OSCAR-generated unique organoid ID. This was performed for a total of over 3,000 cystic (spherical) or budded (irregular) organoids, by matching fluorescently labeled organoid images (Figure 2). We also included organoid morphology and tissue of origin (healthy/tumor) as variables in the model. All factors included in this empirical multiple linear regression model were shown to correlate to a statistically significant degree with the number of cells in an organoid (Figure S3G).

### OSCAR is capable of accurately estimating the number of organoid cells in previously unseen organoid well images

**Figure 3:**
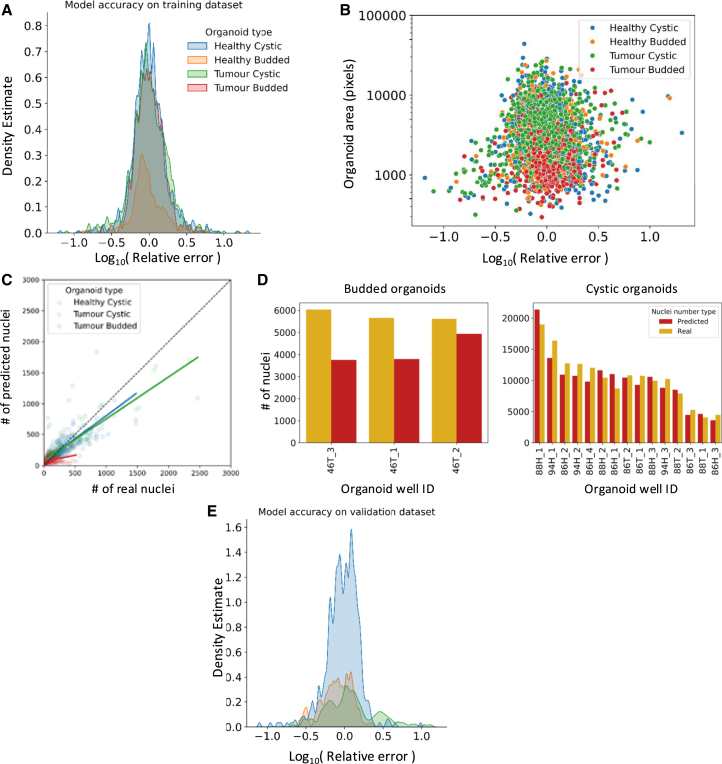
Validating the ability of OSCAR to predict the number of cells within an organoid (A) Kernel density estimate distribution of the log10(relative error), i.e., real number of nuclei divided by estimated number of nuclei, stratified by organoid type. (B) Association between log10(relative error) and organoid area in pixels stratified by organoid type. (C) Pearson correlation between the real number of nuclei and the estimated number of nuclei in the validation dataset, stratified by organoid type. (D) Comparison of real and predicted cell numbers demonstrated per well in the validation dataset. (E) Kernel density estimate distribution of the log10(relative error) of nuclei number predictions in the validation dataset. See also Figures S3 and S4.

We first tested the ability of OSCAR to predict the number of cells in an organoid for images included in the training dataset. We measured accuracy by calculating the relative error, that is, the real number of nuclei in an organoid divided by the number of nuclei predicted to be in that organoid by OSCAR. We took the log10 of each estimate for improved legibility. On the training dataset, OSCAR predicted the number of cells per organoid with a log10(relative error) of 0.1624 (95% CI [0.1569, 0.1678]) (Figures 3A and 3B). Interestingly, when we compared prediction accuracy to the size of the organoid being assessed, the model showed slightly lower accuracy when estimating cell numbers in organoids in the first quartile of area, with a log10(relative error) of 0.1976 (95% CI [0.1841, 0.2111]) (Figure 3B).

To validate the generalizability of our model, we tested the ability of the model to predict the number of cells in organoids that had not been included in the training dataset. To this end, organoid samples from three different patients, which had not been previously seen by OSCAR, were grown as described, and bright-field microscopy images were acquired for each patient sample. The nuclei were then stained to determine the “real cell number.” Each organoid image was passed through OSCAR to obtain an estimate of the area of each organoid and, in turn, an “estimated cell number.” The “estimated cell number” was then compared to the “real cell number” to assess the accuracy of our pipeline on organoids not included in the training data.

We observed a clear positive correlation between the predicted number of nuclei and the real number of nuclei, suggesting only a modest amount of deviation between the estimated and true cell numbers (Figure 3C). To understand how this might impact the estimate of total cell numbers in a well, the real total number of organoid nuclei in all wells was compared to the total estimated number of organoid nuclei (Figure 3D). As with the training dataset, the model estimation on previously unseen organoids had a similar log10(relative error) of 0.1785 (95% CI [0.1661, 0.1909]) (Figure 3E). The estimated number of organoid cells in a well deviated from the real cell number by an average of 16% (95% CI [11%–20%]), showing a degree of accuracy certainly sufficient for use in experimental procedures necessitating the estimate of organoid cell numbers in a well.

### Validation of OSCAR using organoids produced from other tissue types

To ascertain the translatability of OSCAR, we validated its use on organoids derived from pancreatic ductal adenocarcinoma (PDAC). These organoids grow in a cystic fashion, and again, we observed a positive correlation between the number of nuclei predicted by OSCAR and the real number of nuclei (Figures S4A–S4C). The model performed to a similar degree on pancreatic organoids, showing a log10(relative error) of 0.1653 (95% CI [0.071, 0.2591]), suggesting that the models included in OSCAR show an adequate degree of applicability to organoids derived from other tissues, showing similar morphology to colorectal organoids.

### OSCAR generates more consistent estimates of organoid cell numbers than standard methods of organoid cell number estimation

**Figure 4:**
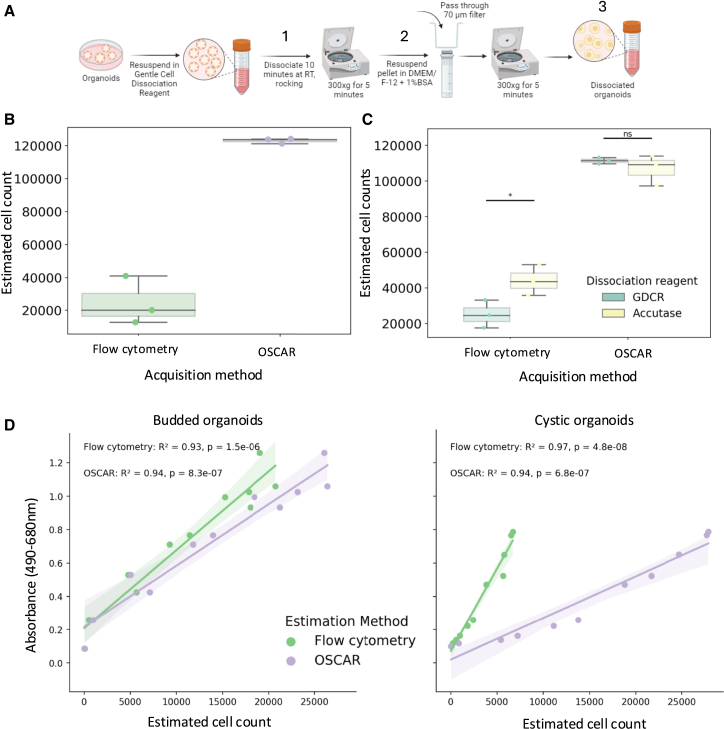
Comparing the cell number estimation to standard cell counting methods To count the number of cells in an organoid, they first need to be dissociated into single cells using a method such as that depicted in (A). “1,” “2,” and “3” denote the steps at which counting was conducted. (B) The computational pipeline consistently counted a higher number of cells with less variation than the flow cytometry method. (C) When comparing Gentle Cell Dissociation Reagent (STEMCELL) and Accutase (Sigma Aldrich) as dissociation reagents, a significant difference in cell count was observed. (D) The number of estimated organoid cells had a strong correlation with the amount of LDH released following organoid lysis, in both cystic and budded organoids, ∗<0.05. No difference was observed when using the computer pipeline. Schematic created using BioRender.com. See also Figure S5.

We sought to compare the performance of OSCAR to other cell counting methods that are currently used. To this end, organoid wells with roughly the same starting number of organoid cells were grown. Once confluency was reached, we estimated the number of cells in each well using OSCAR. Subsequently, we generated an estimate of organoid cell numbers of the same organoids using a flow cytometric approach. To assess variability in cell number estimates generated using flow cytometry, we measured organoid cell numbers at different steps of the procedure (i.e., immediately after dissociation, before a washing step, and after a washing step) (Figure 4A). We observed variability of more than 20,000 cells in the estimated number of organoid cells generated using the flow-cytometry-based approach but observed a high degree of concordance in estimations generated using our computational approach (Figure 4B).

Having shown how the stage at which organoid cells are counted during a dissociation procedure influences the outcome of cell counting using flow cytometry, we sought to assess whether the dissociation reagent used similarly influenced the number of counted cells. We repeated the above procedure with organoid wells plated at the same starting cell number and grown to confluence. These wells were imaged and quantified using OSCAR, after which three wells were dissociated with gentle cell dissociation reagent (GDCR), and three wells were dissociated with Accutase. Dissociated cell suspensions were then used to generate organoid cell counts on a flow cytometer. Accutase-dissociated wells displayed a statistically significant higher cell count than GDCR-dissociated wells, in line with GDCR being marketed as a “gentler” cell dissociation reagent (Figure 4C). In contrast, the cell number estimates generated using OSCAR prior to dissociation did not differ significantly between the two groups. These observations suggest flow-cytometry-based counting is prone to numerous sources of variation, while organoid cell number estimates generated by OSCAR are significantly more consistent.

### Cell number estimates generated by OSCAR correlate with biological measurements

To further scrutinize the cell number estimates generated by OSCAR, we assessed whether its estimates correlated with the release of lactate dehydrogenase (LDH) into a culture well supernatant following complete organoid lysis. To this end, organoids were seeded into 96-well plates at a range of starting cell densities (0–20,000 per well) and grown for 7 days. At this point, all wells were imaged, and cell number estimates for each well were generated using OSCAR. Each well of organoids was then lysed overnight, and LDH measurements were taken using an absorbance-based assay, after which lysed organoid cells were counted using flow cytometry (Figure S5A). In agreement with the accuracy of OSCAR’s estimate described in Figure 3, the number of estimated organoid cells in a well showed a very strong correlation with the amount of LDH released following organoid lysis, something that was true for both cystic and budded organoids (Figure 4D). Flow-cytometry-generated cell number estimates also showed a strong correlation with the amount of LDH released in this assay. However, this required complete organoid lysis since the number of cells counted by flow cytometry varied significantly depending on whether organoids were completely lysed or not (Figure S5A). These experiments show a clear advantage of OSCAR, i.e., the ability to generate biologically relevant organoid cell number estimates from images without the need or variability introduced by organoid pre-processing prior to flow cytometry counting.

### Organoid: Immune cell co-culture assays

**Figure 5:**
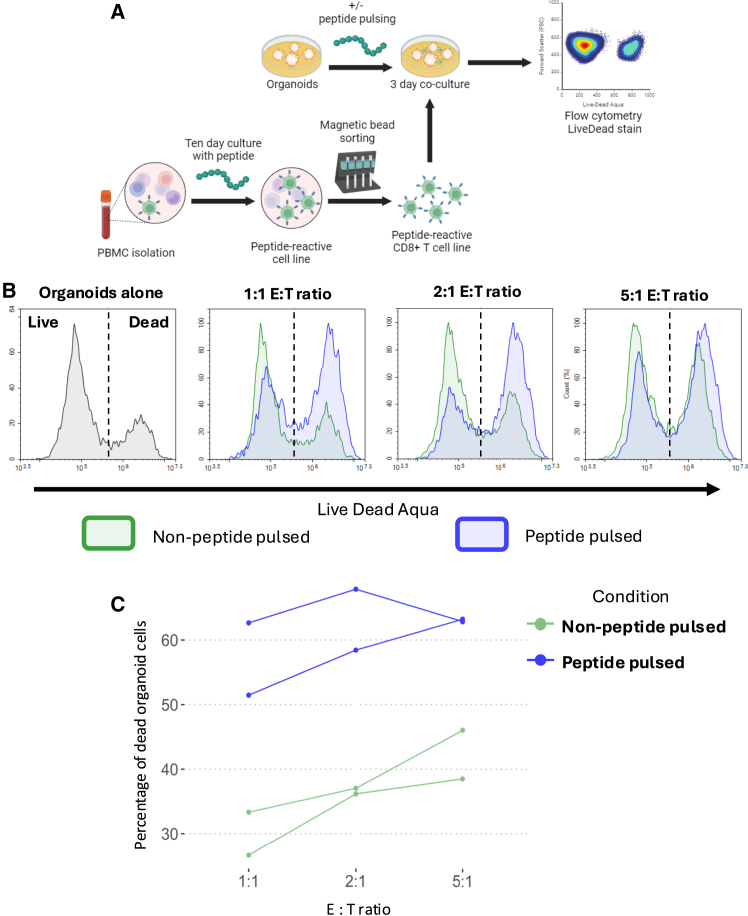
OSCAR enables E:T titrations to be performed during organoid-immune cell coculture experiments (A) Methodological summary of the generation of a peptide-reactive CD8^+^ T cell line and subsequent killing assay performed in coculture with HLA-A2+-matched organoids. (B) Representative live-dead histograms of organoid cells cocultured with different E:T ratios. (C) Percentage of dead organoid cells cocultured with different E:T ratios under non-peptide-pulsed or peptide-pulsed conditions. E:T = Effector:Target. Schematic created using BioRender.com. See also Figure S5.

Given the rising interest in using organoids for organoid-immune cell co-culture assays,^10^^,^^11^ we sought to assess the feasibility of using OSCAR in this context. We set up a tumor organoid-T cell co-culture at different E:T ratios (Figure 5A). To achieve this, we generated peptide-reactive T cell lines from an HLA-A2^+^ donor and grew an HLA-A2^+^ tumor-derived organoid (Figure S5C).

We calculated the E:T ratio for different conditions by imaging and estimating the number of organoid cells in each well using OSCAR prior to organoid dome disruption. We pulsed organoid cell suspensions with the relevant peptides for 1 h at 37°C, after which we washed the organoids to remove any unbound peptide. By co-culturing immune cells with organoids after peptide pulsing in this fashion, organoids were able to act as antigen-presenting cells and induce a CD8^+^ T cell response (Figure S5D). We next performed co-cultures at different E:T ratios (1:1, 2:1, and 5:1), the ratios being calculated using organoid cell number estimates generated by OSCAR. Following 72-h incubations, we observed consistently higher organoid death in organoids that had been peptide-pulsed and co-cultured with CD8^+^ T cells than those that had not been peptide-pulsed (Figures 5B, 5C,S5D, and S5E). The degree of background killing increased with higher numbers of effector T cells, obscuring measurement of peptide-specific killing. These data highlight the need for accurate cell measurements to facilitate reproducible estimates of E:T cell ratios.

As such, we show that OSCAR can be employed in the workflow of organoid-immune cell co-cultures to generate organoid cell estimates that contribute to generating consistent experimental results.

## Discussion

We have created OSCAR, a computational pipeline that generates an estimate of the cell numbers within an organoid culture well solely using a bright-field microscopy image taken at 4× magnification. The use of this pipeline will allow for faster estimation of cell numbers and reduce the waste associated with dissociation and flow cytometry counting of cells.

We exhibit the applicability of OSCAR by testing it on organoid samples that were not included in the training data and showed that OSCAR displayed strong robustness, capable of estimating the total number of organoid cells in a culture well, with a 16% deviation from the real number of cells, showing a degree of accuracy certainly applicable to organoid-based assays (Figure 3). We also showed that OSCAR was capable, with a similar degree of accuracy, of estimating the number of PDAC organoid cells despite being trained on colorectal organoids, indicative of OSCAR’s translatability to other organoid models. We do, however, encourage users who wish to use OSCAR on organoid samples distinct from intestinal and pancreatic organoids to perform simple validation experiments like those described in Figures 3A–3D, since organoids with distinct structures, such as those that lack a lumen, are likely to comprise significantly different cell densities. Next, we demonstrated that OSCAR is capable of generating organoid cell number estimates that correlate significantly with the LDH concentration in the supernatant during an LDH-absorbance-based assay (Figure 4). Furthermore, we successfully applied OSCAR in an organoid-T cell co-culture assay, which demonstrated the importance of titrating E:T ratios to enable assessment of peptide-specific killing (Figure 5).

Currently, several methods are routinely used to generate organoid cell number estimates when setting up organoid-based assays: (1) when harvesting a well, half of it is dissociated and counted manually to estimate cell number in the remaining half; (2) an extra well of organoids is grown to dissociate and count manually, which is then used as an estimate of the number of cells in the remaining wells; and (3) examining the wells by eye and judging the organoids by size before experimentation. When testing flow-cytometry-based methods, we found their estimates to be variable, time-consuming, and wasteful of both organoids and consumables. OSCAR was developed to overcome such material waste and to ensure cell number estimates showed a lesser degree of variation. We highlight to users that the cell number generated by OSCAR is an estimate, and as shown in Figures 4C and 4D, OSCAR generates cell number estimates that are higher than those generated by flow cytometric approaches. This is likely to be due to a combination of (1) incomplete organoid dissociation, (2) organoids remaining in the culture well during pre-processing/cell loss during filtering stages, and (3) a degree of overestimation by OSCAR. While both flow-cytometry- and OSCAR-generated cell number estimates correlated significantly with biological measurements, we have shown that flow cytometric counting is prone to pre-processing induced variability (Figures 4B and 4C; Figure S5B), something that is virtually absent from OSCAR, with the added benefit of reduced material and organoid waste. Another major advantage of using OSCAR over other methods, such as single-cell quantification in organoids,^5^^,^^12^^,^^13^ is that our system is label-free, and the organoids can be monitored throughout culture, rather than an endpoint assay requiring fixation. While OSCAR is in its first iteration, which we aim to continue to update and improve, we have shown that OSCAR allows us to take a simple bright-field image of a well of organoids and generate consistent estimates of organoid cell numbers without dissociating or disturbing the organoids in any way. We make OSCAR freely available to use for the organoid research community here: https://colab.research.google.com/drive/1paRiDvvAu4ezZEesSdUH-fO_BJ8eJgsY?usp=sharing.

### Limitations of the study

OSCAR is not without limitations. Given that its estimate is based primarily on organoid area within an image, it cannot take into account the presence of dead cells within organoid cultures. Similarly, OSCAR may be inadequate for use when factors that alter cell density are at play. For example, certain toxins are known to alter the size of organoid lumen, which would introduce significant inaccuracies into cell number estimates.^14^ Furthermore, we have observed that organoids shrink when in co-culture with activated CD8^+^ T cells, likely owing to ongoing cell death (unpublished data). In such instances, OSCAR’s use for monitoring organoid cell number would be inaccurate. Given these limitations, the quantification provided by OSCAR should be used as an estimate that can aid in monitoring organoid growth and in experimental planning. Future iterations of OSCAR may want to consider other organoid factors, such as organoid perimeter and the ratio of organoid area to organoid perimeter, when predicting organoid cell numbers.

## Resource availability

### Lead contact

Further information and requests for resources and reagents should be directed to and will be fulfilled by the lead contact, Awen M. Gallimore (gallimoream@cardiff.ac.uk).

### Materials availability

This study did not generate new unique reagents.

### Data and code availability

• Image data have been deposited in OSF (DOI https://doi.org/10.17605/OSF.IO/EHDTK) and are publicly available as of the date of publication. The DOI is also listed in the key resources table.
• All original code is also deposited in OSF (DOI https://doi.org/10.17605/OSF.IO/EHDTK) and is publicly available as of the date of publication. A GitHub repo is available to raise any issues at https://github.com/LCapitani/OSCAR.
• Any additional information required to reanalyze the data reported in this work paper is available from the lead contact upon request.

## Acknowledgments

We thank the Wales Cancer Biobank for assistance with the consent of patients and the collection of colorectal tissue. We also thank the authors of the publication of OrgaQuant for making their image datasets freely available to the community.

The laboratory is supported by awards from The 10.13039/100010269Wellcome Trust 209213/Z/17/Z and 10.13039/501100000289CRUK DRCRPG_NOV21/1000003. S.E.A.B. is funded by the 10.13039/100018601Wales Cancer Research Centre (10.13039/100018601WCRC). L.C. is funded by GW4 BIOMED 10.13039/501100000265MRC DTP from the 10.13039/501100000265MRC/10.13039/100014013UKRI. C.A.H. is funded by a 10.13039/501100000849National Centre for the Replacement, Refinement and Reduction of Animals in Research (NC3Rs) PhD studentship. L.M.B. is funded by a 10.13039/501100000289Cancer Research UK Biotherapeutic Program grant to A.L.P. (reference C52915/A29104).

## Author contributions

S.E.A.B. and L.C. conceptualized the project. S.E.A.B. and K.W. consented the patients. S.E.A.B. developed organoids and carried out imaging and analysis. L.C. developed the code for OSCAR. L.C., S.E.A.B., and C.A.H. generated organoid annotations for model training. S.E.A.B., L.C., C.A.H., and Y.C. carried out biological experiments (including LDH and co-culture). L.M.B. and A.L.P. provided cultured pancreatic organoids for validation. A.M.G. and A.J.G. acquired funding and supervised the project. All authors reviewed and edited the manuscript.

## Declaration of interests

The authors declare no competing interests.

## STAR★Methods

### Key resources table

**Table:** 

REAGENT or RESOURCE	SOURCE	IDENTIFIER
**Antibodies**		

FITC Anti-human IFNγ	BioLegend	Cat# 506504, RRID:AB_315437
Mouse PE/Cyanine7 Anti-human TNFα	BioLegend	Cat# 502930, RRID:AB_2204079
Mouse APC Anti-human CD3	BioLegend	Cat# 300412, RRID:AB_314066
Mouse Brilliant Violet 421 Anti-human CD4	BioLegend	Cat# 300532, RRID:AB_10965645
Mouse PE/Dazzle 594 Anti-human CD8	BioLegend	Cat# 344744, RRID:AB_2566515
Anti-human HLA-I	BioLegend	Cat# 311430, RRID:AB_2561617
Anti-human HLA-A2	BioLegend	Cat# 343308, RRID:AB_2561567

**Biological samples**		

Human colorectal tissue for patient derived organoids (PDOs)	Wales cancer bank, Cardiff, UK	N/A
Human peripheral blood mononuclear cells (PBMCs)	Cardiff University, Cardiff, UK	N/A

**Chemicals, peptides, and recombinant proteins**		

MACS tissue storage solution	Miltenyi Biotec	Cat# 130-100-008
Antibiotic-Antimycotic (100X)	Gibco	Cat# 15240062
Gentle Cell Dissociation Reagent	STEMCELL Technologies	Cat# 100-0485
Advanced DMEM/F-12	Gibco	Cat# 12634010
Bovine Serum Albumin (BSA)	Sigma	Cat# A9418
Matrigel Growth Factor Reduced	Corning	Cat# 356231
IntestiCult Organoid Growth Medium	STEMCELL Technologies	Cat# 06010
Penicillin-Streptomycin (10,000 U/mL)	Gibco	Cat# 15140122
Rock inhibitor Y-27632	STEMCELL Technologies	Cat# 72307
Lymphoprep	STEMCELL Technologies	Cat# 18061
RPMI	Gibco	Cat# 3187-025
GlutaMAX	Thermo Scientific	Cat# 35050061
Sodium Pyruvate	Gibco	Cat# 11360070
RBC lysis buffer 10X	BioLegend	Cat# 420301
CTL Test Plus	ImmunoSpot	Cat# CTLTP-005
Hoechst 3342, trihydrochloride trihydrate	Invitrogen	Cat # 11534886
CEF extended (CD8) human PepPool	MABTECH	Cat# 3618-1
IL-2	Proleukin, from University Hospital Wales Pharmacy, Cardiff, UK	Cat# 73776-0022-01
IL-15	R&D Systems	Cat# BT-015
Penicillin/streptomycin/amphotericin	Thermo Scientific	Cat# 15240062
Accutase	Thermo Scientific	Cat# 00-4555-56
Brefeldin A	Sigma	Cat# B5936
Live/Dead Aqua	Thermo Scientific	Cat# L34957
Rat serum	Invitrogen	Cat # 31888
Permeabilisation solution	BD Biosciences	Cat# 554655
Fixation buffer	BD Biosciences	Cat# 554722

**Critical commercial assays**		

CytoQUANT LDH assay	Invitrogen	Cat# C20300

**Deposited data**		

Image and annotation data	This paper	OSF, DOI: https://doi.org/10.17605/OSF.IO/EHDTK

**Experimental models: Cell lines**		

(ATCC® PDM30™) Pancreatic ductal adenocarcinoma (PDAC) organoid line	ATCC	HCM-CSHL-0079-C25
(ATCC® PDM38™) Pancreatic ductal adenocarcinoma (PDAC) organoid line	ATCC	HCM-CSHL-0091-C25
Original code for OSCAR	This paper	OSF, DOI: https://doi.org/10.17605/OSF.IO/EHDTK; https://github.com/LCapitani/OSCAR, Tool: https://colab.research.google.com/drive/1paRiDvvAu4ezZEesSdUH-fO_BJ8eJgsY?usp=sharing
Imaris	Oxford Instruments, Zurich	RRID:SCR_007370
Python	Python Software Foundation	RRID:SCR_008394
Pandas (python library) version 1.3.5	McKinney^15^; pandas development team, 2020	https://pandas.pydata.org
Scikit-learn (Python library) version 1.0.2	Pedregosa et al.^16^	https://scikit-learn.org
FlowJo v10	BD Biosciences	RRID:SCR_008520
VGG Image Annotator	Dutta and Zisserman,^17^ Oxford University, UK	https://www.robots.ox.ac.uk/∼vgg/software/via/
Mask R-CNN	He et al.^7^; Matterport GitHub	https://github.com/matterport/Mask_RCNN

### Experimental model and study participant details

#### Organoids

##### Organoid lines

Tissue samples for the generation and growth of colorectal organoids were acquired during surgical resection at the University Hospital Wales (UHW), Cardiff, UK, under ethics IRAS Project ID: 179819. Informed consent was obtained prior to sample collection.

For further validation, we used models and data derived by the Human Cancer Models Initiative (HCMI) https://ocg.cancer.gov/programs/HCMI; dbGaP accession number phs001486. Pancreatic organoids HCM-CSHL-0079-C25 (ATCC PDM-30) and HCM-CSHL-0091-C25 (ATCC PDM-38) were purchased from the ATCC repository and grown according to the supplier’s instructions.

##### Derivation of patient-derived organoids (PDOs)

Normal and tumor tissues were either processed immediately following resection, or after a maximum of 24 h in MACS tissue storage solution. Crypts were extracted from the tissues as follows: Normal and tumor colorectal tissue was washed twice with ice-cold PBS supplemented with penicillin (100 units/mL), streptomycin (100 μg/mL) and amphotericin B (0.25 μg/mL) (pen/strep/amph). The tissue was then transferred to a 10 cm^2^ Petri dish and minced into fragments using a scalpel. The tissue fragments were transferred to a 15 mL tube and washed twice with ice-cold PBS supplemented with pen/strep/amph. The tissue was dissociated using the Gentle Cell Dissociation Reagent (GCDR) for 30 min, rocking on ice. Following this, the crypts were isolated by vigorous pipetting in Advanced DMEM/F-12 with 1% BSA and passing the tissue through a 70 μm cell stainer. The crypts were counted, at least 2000 crypts/dome were resuspended in DMEM/F-12 + 1% BSA, and Matrigel was added in a 1:1 ratio before being plated in a 24-well plate, in a 50 μL dome. The plates were incubated at 37°C, 5% CO2 for 30 min to allow for solidification of the Matrigel. Following this, Intesticult Organoid Growth Medium supplemented with penicillin and streptomycin (pen/strep), and Y-27632 was added at 500 μL/well. Media was replaced every 2–3 days; organoids were passaged once confluent.

For model training and validation, 19 different patient-derived organoid lines were used. For validation, only organoids that were not part of the original training dataset were assessed. These organoids vary in origin (tumor or healthy), morphology (cystic or budded), age and sex as outlined in Table 1. Factors like sex and age were not included in the model to make the model as generalised as possible.

**Table 1:** Patient-derived organoid characteristics used in the training and validation of the model

Patient no.	Sex	Age (y)	Origin tissue	Healthy or tumor	Organoid morphology	Used for
SBWCRC007	M	84	ascending colon	healthy	cystic	training data
				tumor	cystic	training data
SBWCRC016	M	74	sigmoid colon	tumor	budded	training data
SBWCRC025	M	62	sigmoid colon	tumor	budded	training data
SBWCRC033	M	72	rectal	healthy	cystic	training data
				tumor	cystic	training data
SBWCRC035	M	64	sigmoid colon	tumor	cystic	training data
SBWCRC046	F	66	splenic flexure	tumor	budded	training data and model validation
SBWCRC050	M	58	sigmoid colon	healthy	cystic	training data
				tumor	cystic	training data
SBWCRC054	F	60	caecal	tumor	budded	training data and functional validation (co-culture)
SBWCRC065	M	67	rectal	healthy	budded	training data
SBWCRC067	M	68	caecal	healthy	cystic	training data
SBWCRC068	M	81	rectal	healthy	cystic	training data
				tumor	cystic	training data
SBWCRC069	M	83	ascending colon	tumor	budded	training data
SBWCRC070	F	79	caecal	healthy	cystic	training data
SBWCRC071	M	82	sigmoid colon	tumor	budded	training data
SBWCRC076	M	73	ascending colon	healthy	budded	training data
				tumor	budded	training data
SBWCRC082	F	65	rectal	tumor	budded	training data
SBWCRC086	M	52	splenic flexure	healthy	cystic	training data and model validation
				tumor	cystic	training data and model validation
SBWCRC088	M	77	sigmoid colon	healthy	cystic	training data and model validation
				tumor	cystic	training data and model validation
SBWCRC094	M	65	transverse colon	healthy	cystic	training data and model validation

For experimental validation of the model, HLA-A∗02:01 homozygous tumor organoids from patient SBWCRC054 were used for HLA-matched co-culture with allogenic PBMC. This experiment served as a proof of concept and functional validation of the computational pipeline. Therefore, only one well-characterised organoid line was utilised as prior extensive validation of multiple organoids had already taken place.

##### Maintenance of PDOs

Organoids were passaged once cultures reached confluency. A 24-well plate was pre-warmed at 37°C, and Matrigel was thawed on ice. Medium was aspirated from the organoid wells, and cultures were treated with GCDR. Organoids were dislodged by gentle pipetting, collected and incubated at room temperature for 10 min, rocking. Suspensions were centrifuged for 5 min to pellet, and the supernatant was discarded. Pellets were washed once with ice-cold DMEM-F-12 + 1% BSA, filtered through a 70 μm cell stainer, and centrifuged again. The final pellet was resuspended in a 1:1 (v/v) DMEM/F-12 + 1% BSA and Matrigel and seeded into a pre-warmed 24-well culture plate in domes. Domes were left to set for 30 min at 37°C, 5% CO2, before adding Intesticult Organoid Growth medium.

#### Human PBMC culture

Recruitment of healthy adult volunteers was approved by Cardiff University’s School of Medicine Research Ethics Committee under ref. 18/04. For the co-culture experiment, HLA-A∗02:01-positive PBMC was taken from a single healthy male donor (28 yo). The experimental outcome depends on HLA-restricted antigen recognition, rather than donor demographic factors.

Peripheral blood was obtained from a consented HLA-A∗02:01 positive healthy donor in 10 mL heparinised vacutainers. Peripheral blood mononuclear cells (PBMCs) were isolated by density gradient centrifugation using Lymphoprep. Briefly, 20 mL of Lymphoprep was prepared in a fresh 50 mL Falcon tube, and the blood was carefully layered on top. Samples were centrifuged at 2000 rpm for 20 min at room temperature, with the brake off (acceleration = 2, deceleration = 1). The PBMC layer was collected using a Pasteur pipette and transferred into a fresh 50 mL tube, and R+ media (RPMI supplemented with GlutaMAX, sodium pyruvate, and pen/strep) was added to achieve a final volume of 40 mL, followed by centrifugation at 2000 rpm for 10 min at room temperature (acceleration = 9, deceleration = 9). The supernatant was discarded, and if the pellet appeared red, 5 mL of RBC lysis buffer was added for 5 min. The reaction was quenched by adding 20 mL of R+ media, followed by centrifugation at 1600 rpm for 5 min at room temperature (acceleration = 9, deceleration = 9). The pellet was resuspended in 10 mL RPMI, and flow cytometry-mediated cell counts were performed. Cell suspensions were adjusted to seed 200,000 PBMCs per well in CTL-Test PLUS medium, supplemented with GlutaMAX and pen/strep, adding cytokines and peptides as required. The cells were incubated at 37°C, replacing half the media when required.

### Method details

#### Design of model

##### Dataset sources and pre-processing

Model training in the context of instance segmentation models relies on the developers providing a dataset of images in which the objects that should be detected (i.e., organoids) have been manually correctly labeled (e.g., the outline of all organoids in an image highlighted). To generate such a dataset, we compiled images from two sources. These were:

A subset of colorectal organoid images made publicly available with the previously published (now no longer supported) organoid detection tool OrgaQuant. The images in this publication were published for the development of an object detection model rather than an instance segmentation model - as such, they were not made available with corresponding masks (outline labels).

A subset of colorectal organoid images derived from patients acquired in-house on a bright-field microscope (EVOS XL Core, Thermo Scientific) at 4× magnification. These images were acquired at various stages of organoid growth to contribute to the generation of a robust detection model.

From these, features of interest (i.e., organoids) were labeled utilising the software VGG Image Annotator, which is made freely available by the Visual Geometry Group at Oxford University (https://www.robots.ox.ac.uk/∼vgg/software/via/).

##### Dataset augmentation

Given the context of the images (i.e., organoids), we employed image augmentation procedures that would train a model capable of recognising features typically observed in bright-field microscopy images (Figure 1A). These were

• Horizontal and vertical flipping of images to ensure the model was able to recognise organoids irrespective of orientation.
• Each image in the dataset underwent a random 'zoom-in' using a cropping augmentation to ensure that the model was effective at a range of organoid sizes.
• A blur augmentation was added to randomly blur each image. This augmentation aimed to mimic organoids, which show up in bright-field images but are found below/above the focal plane of the image. The aim here was to ensure that a subset of the images in the dataset was blurred enough to contribute toward the partial recognition of blurred organoids.
• Each image was exposed to a randomised brightness and contrast alteration to alter the image brightness and contrast. This was crucial, given that organoid images acquired with a bright-field microscope often suffer from regions that are overly bright or dark and regions that have high levels of contrast.

Each image in the dataset was exposed to the above augmentations during each round of training.

##### Bright-field imaging of whole organoid wells

To image an entire organoid well, we utilised a bright-field microscope with a camera (EVOS) at 4× magnification. Wells were then imaged in a 2x3 quadrant (a total of 6 images per well) with overlapping borders as shown in Figure 1B. We then employed the OpenCV2 Stitcher Class in Python in our analysis pipeline to allow these overlapping images to be stitched into a single image for downstream analysis.

##### Instance segmentation model development

We modified the published Mask R-CNN architecture^7^ to enable its use on 2-dimensional arrays, thereby reducing the size of input data from 3-channelled colored images to 1-channelled images in black and white, significantly reducing processing time, with separate models for either cystic or budded organoids (Figure 1C).

The application of the above process outputs an image depicting an entire organoid culture well with organoids identified as shown in Figure 1D. For later steps of our pipeline, a CSV file containing the area of each organoid is generated.

##### Mosaicking algorithm

The mosaicking approach employed is not as simple as splitting an image into sections, applying the model to each section and then combining the results. The difficulty in this approach stems from the fact that some objects of interest (organoids) may fall between the boundaries of two tiles (Figures S1A and S1B). If analyzed in this fashion, then the object of interest would be divided into two separate objects in the final output. To overcome this, a sliding window was employed with a stride dimension of 256 pixels (i.e., half of the length of one tile, 512). In essence, an image is split into tiles of 512x512 pixels starting in the top-left corner of the image at coordinates (0,0). A step of 256 pixels is then taken in the x-dimension, where the next tile is generated with starting (x,y) coordinates of (256,0). The sliding windows then continues to stride across the image until the image boundary is met, after which a 256-pixel step is taken in the y-dimension, where the process is repeated. See Figure S1 for a visual depiction of this process.

If one considers a hypothetical image of 1024x1024 pixels, if it were to be made into tiles of 512x512 pixels without employing a sliding window, this would generate 4 tiles. However, if a sliding window with 256-pixel strides is employed, this generates 9 overlapping images to which the model can be applied. This ensures that every organoid occurs at least once in the middle of a tile as opposed to its edge. (Figure S1B).

This, however, generates a new problem where objects of interest may be detected more than once if they fall within multiple tiles, as shown in Figure S1A. To overcome this, a modified version of the no-maximum suppression algorithm described in the aforementioned research paper on satellite images was employed.^9^ The steps taken by this algorithm, shown in Figures S2A–S2C, are as follows:

1. Take tile X in the center of an image. Based on its coordinates within the image, adjacent tiles are identified.
2. Then each identified object in tile X is compared to every object in adjacent tiles to see if they overlap.
3. Objects in different tiles that are found to overlap are then investigated further to assess whether they represent the same organoid. To do so, the ratio of the overlapping area to the size of each object is calculated. If the two objects overlap by more than 30% then they are considered to be the same organoid.
4. In this instance, the detected objects are sorted by size, and the one with the largest area is kept in the tile where it belongs, whilst the one with the smallest area is deleted from its tile.

##### Empirical model development

To develop this model to relate the number of organoid cells in an organoid to its estimated area, organoids were grown to confluency and harvested using PBS, washed once with DMEM/F-12 + 1% BSA and reseeded into IBIDI μ-Slide 8 Well plates (IBIDI, cat# 80800) as above. Plates were incubated at 37°C, 5% CO_2_ for at least 24 h before being imaged using an EVOS bright-field imager (ThermoScientific) at 4× magnification. The Mask R-CNN model developed above was then employed on these images to detect organoids and estimate the area of each detected organoid.

Following this, organoids were stained with Hoechst 33342, 1:1000 for 30 min at 37°C, 5% CO2. The media was replaced, and z-stacks of the organoids were obtained using a confocal microscope (Zeiss LSM800 confocal laser scanning microscope). These z stack images were opened using Imaris and viewed using 3D View. Then, using the Spots selection tool, each nucleus was detected by using Hoechst as the source channel and an estimated XY diameter between 7 and 10 μm. Each image was finalised by manual visual confirmation, after which the spot quantification was performed to count how many spots (i.e., nuclei) belonged to each organoid.

Cystic and budded organoids in fluorescently labeled images were then matched to their respective bright-field images, and using the unique organoid ID given to every organoid detected by OSCAR, the number of nuclei in an organoid was matched to its area. This workflow is visually described in Figure 2. The dataset generated by this combined effort contains over 3000 organoids for which the following information was characterised:

1. Organoid area in image (in pixels).
2. Estimated number of nuclei in organoid.
3. Organoid tissue origin (i.e., healthy or tumor).
4. Organoid morphology (i.e., cystic or budded).

#### Biological validation

##### LDH assay

Organoids were cultured until confluent, and several wells were imaged and counted using OSCAR. Based on these counts, seeding density was calculated to yield 660,000 cells across 66 wells. An 11-point dilution series (20,000 to 0 cells, in 2,000-cell intervals) was prepared in six replicates. Organoids were seeded in 50 μL layers of 50% Matrigel, allowed to set for 30 min, and topped with 200 μL of IntestiCult medium. The organoids were cultured for 7 days, with a 150 μL media change on day 3. The CyQUANT LDH Cytotoxicity Assay was used in subsequent steps.

On day 7, organoids in the first triplicate rows (33 wells) were imaged using OSCAR, lysed with 15 μL of 10X Lysis Buffer, and incubated overnight. The remaining wells were imaged the next day. Supernatants (50 μL) from each well were transferred to a new 96-well plate, and 50 μL of the reaction mixture was added. After 45 min of incubation at room temperature, 50 μL of stop solution was added, and absorbances were measured at 490 nm and 680 nm using a CLARIOstar plate reader (BMG Labtech). Organoids from the original plate were dissociated using Accutase, and cell suspensions were analyzed by flow cytometry with live/dead staining. LDH production was calculated by subtracting absorbances and compared to cell counts from both OSCAR and flow cytometry to assess correlations.

#### Flow cytometry

In this study, both organoids and PBMC underwent flow cytometry staining. Organoids were dissociated into single cells with Accutase or GCDR for 30 min and quenched with DMEM/F 12 + 1% BSA.

Organoids and PBMC were pelleted at 1600 rpm for 5 min. Cells were stained with Live/Dead Aqua (1:1000) for 30 min at room temperature, washed in FACS buffer (PBS +2% FBS), and, where appropriate, were blocked with rat serum (1:50) for 10 min. Surface staining was performed with anti-human pan HLA-A, B, C and anti-human HLA A2 or anti-human CD3, anti-human CD4, and anti-human CD8 antibodies diluted 1:20 in FACS buffer for 20 min at room temperature. After washing, cells were resuspended in 100 μL FACS buffer and analyzed on a NovoCyte (Agilent Technologies) flow cytometer. Data were processed in FlowJo v10.

To confirm PBMC reactivity to CEF prior to organoid co-culture, intracellular cytokine staining was performed. On day 10 post stimulation, three to five wells of PBMC were randomly selected, gently mixed, and counted. The selected wells were pooled, centrifuged at 1,600 rpm for 3 min, and the pellet was resuspended in 50 μL PBS and washed three times. The cells were separated into new wells (100 μL each) for stimulation with different concentrations of CEF expanded human CD8 peptide pool (0.5 μL, 1 μL, and a PBS negative control). 0.2 μL of brefeldin A was added to all stimulation conditions. Cultures were supplemented with 50 μL medium and incubated for 5–6 h or overnight at 37°C.

Following stimulation, cells were centrifuged for 5 min and stained with Live/Dead Aqua dye and surface-stained with anti-CD3, anti-CD4 and anti-CD8 antibodies as above. Cells were washed twice with FACS buffer, stained with the antibody cocktail and fixed overnight in 100 μL fixation buffer. The following day, the cells were centrifuged, and a permeabilisation buffer was prepared (1 mL in 4 mL FACS buffer). The cells were washed with 100 μL of the working permeabilisation solution. Intracellular staining for PE-Cy7 anti-human TNFα and FITC anti-human IFNγ antibodies was performed by resuspending cells in 30 μL of permeabilisation solution containing 1 μL of each antibody, followed by incubation for 30 min at 4°C. Finally, cells were washed and resuspended in 50 μL FACS buffer for flow cytometry analysis.

#### Organoid T cell co-culture

To test the applicability of the cell counting pipeline, OSCAR was used to investigate the ability of organoids to stimulate T cells at different effector-to-target ratios of tumor cells and T cells. PBMCs from a healthy HLA-A2^+^ donor were pulsed with a 10 mg/mL final concentration of the CEF peptide pool, containing HLA-A2-restricted T cell epitopes from cytomegalovirus, Epstein-Barr virus and influenza virus and grown for 10 days, with addition of 20 IU/mL IL-2 and 25 ng/mL IL-15 to the culture media on Day 1 of culture. On day 10, CEF-specific reactivity was confirmed by performing intracellular IFN-γ cytokine staining (see flow cytometry). HLA-A2^+^ organoids were imaged and counted using OSCAR, and the cell number was established before harvesting using PBS and centrifugation at 300*g* for 5 min to pellet. The cells were washed with DMEM/F-12 + 1% BSA to prevent clumping. The pellet was resuspended in 1 mL of organoid media, and this was then split into two 500 μL tubes. To one tube, 10 μg/mL of the CEF peptide pool was added, incubated at 37°C for 1 h, pelleted and washed thrice in PBS to remove any unbound peptides before being resuspended in media. The number of T cells required was determined, and the organoids and T cells were seeded together in 25% Matrigel and incubated for 24–72 h. The organoids were then dissociated using Accutase, and the cell suspension was analyzed by flow cytometry.

### Quantification and statistical analysis

Unless stated otherwise, statistical analysis was performed using a two-sided independent *t* test.

The above data for over 3000 organoids was the input for the multiple linear regression model. Modeling was performed in Python using the Pandas library for data frame manipulation and the scikit-learn library for the machine learning employed. Additional details of data transformations employed to meet modeling assumptions are described in Figures S3A–S3F.

For model development, the linear model function from the scikit-learn library was employed to generate the multiple linear regression model utilising the following model formula:

log(Cellnumber)∼log(Organoidarea)+Sampletype+Morphológy

denoting the cell number as the dependent variable. Sample type denotes whether the sample originates from healthy or tumor tissue, whilst morphology describes the budded/cystic nature of the organoid sample. All factors included in the model were shown to correlate to a statistically significant degree with the number of cells in an organoid (Figure S3G).

#### Assessing the predictive value of organoid perimeter and organoid area to perimeter ratio

We sought to understand whether the inclusion of organoid perimeter and the ratio of organoid area to its perimeter into OSCAR offered any improvements in accuracy. To test this, we randomly selected 7 organoid wells containing a total of 145 organoids for which we calculated the organoid area, organoid perimeter, as well as the ratio of organoid area to organoid perimeter.

Notably, we observed that area, perimeter and the ratio of the two positively correlated with the number of nuclei, albeit to a lower degree than in our larger cohort, likely owing to the smaller sample size employed (Figure S3H). Organoid perimeter showed the strongest correlation with the number of nuclei by a negligible margin. Importantly, we observed that organoid area and organoid perimeter showed a very strong correlation of r-squared 0.95. As with our larger cohort, organoids with budded morphology and tumor organoids (both encoded by the number 1) had a reduced number of nuclei. Since we observed similar trends to our larger cohort, we proceeded to model the number of nuclei using organoid area, perimeter, area to perimeter ratio, morphology and sample type as predictors. We report model accuracy as relative error, equal to the real number of nuclei divided by the predicted number of nuclei, reporting the log10(relative error) for improved legibility.

Using this model, which included all variables, our model performed with an absolute relative error of 0.200, 95% CI [0.165,0.235]. We then tested how removing organoid perimeter from the model influenced accuracy, employing a model like that described in the main paper, i.e., using only organoid area, morphology and sample type to predict organoid nuclei number. We observed a negligible reduced accuracy relative to the model which included organoid perimeter, observing an absolute relative error of 0.202, 95% CI [0.166, 0.238]. The lack of increase in accuracy of the model with added parameters may have been due to the high degree of collinearity between organoid area and organoid perimeter.

### Additional resources

We have released OSCAR for use here: https://colab.research.google.com/drive/1paRiDvvAu4ezZEesSdUH-fO_BJ8eJgsY?usp=sharing.

## Supplemental information

Document S1. Figures S1–S5

Document S2. Article plus supplemental information
