# Supplementary material for: OSCAR is an online ML-powered tool for organoid cell counting using bright-field images
Source: Cell Rep Methods. 2025 Dec 2;5(12):101251. doi: 10.1016/j.crmeth.2025.101251 (PMC12859458; doi:10.1016/j.crmeth.2025.101251)
Supplement: Document S1. Figures S1–S5 [file mmc1.pdf]

Cell Reports Methods, Volume *5*

Supplemental information

OSCAR is an online ML-powered tool for organoid cell counting using bright-field images

Stephanie E.A. Burnell, Lorenzo Capitani, Chloe A. Harris, Luned M. Badder, Alan L. Parker, Kasope Wolffs, Yuan Chen, Andrew J. Godkin, and Awen M. Gallimore

Supplementary

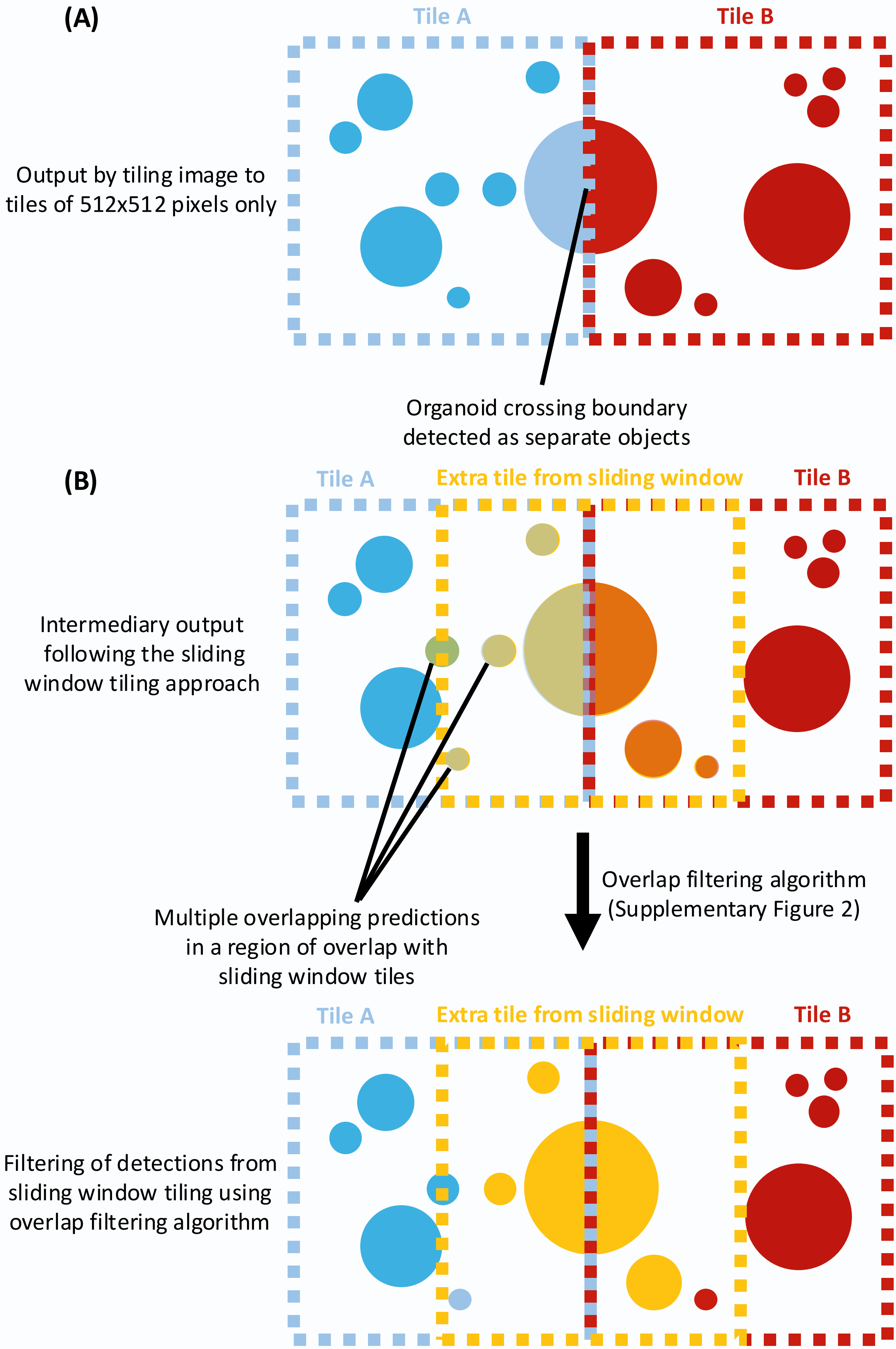

Supplementary Figure 1: Tiling algorithm summary (A) In a tile-based approach, objects at the intersection of two tiles are detected as two distinct objects. (B) Using a sliding window-based approach combined with an overlap selection algorithm as described in Supplementary Figure 2 can overcome this issue.

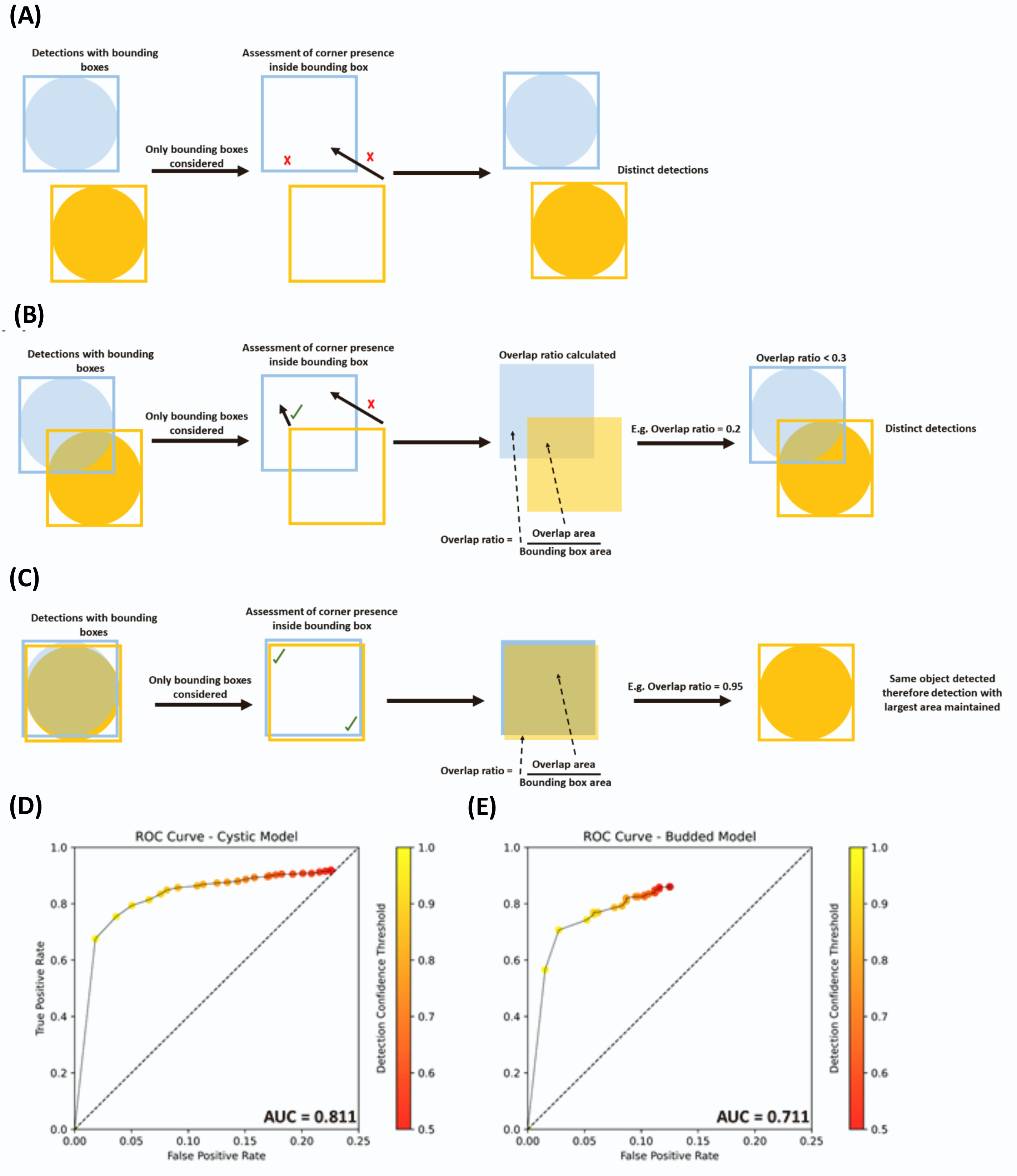

Supplementary Figure 2: Overlap detection algorithm summary and MaskRCNN models accuracies (A) If the corner of object A's bounding box is outside of object B's bounding box, the two objects are considered non-overlapping. (B) If object A's bounding box is contained within object B's bounding box, the overlap ratio is computed as the overlap area divided by the area of object A's bounding box. If this overlap area is less than 0.7, the objects are classified as distinct. (C) If the overlap area is equal to or greater than 0.7, the objects are deemed to be the same. In this case, the object with the larger area is retained. (D) ROC curve of cystic model, showing model true positive and false positive rates at different confidence thresholds, with an AUC of 0.811. (E) ROC curve of budded model, showing model true positive and false positive rates at different confidence thresholds, with an AUC of 0.711. Related to Figure 1.

Supplementary

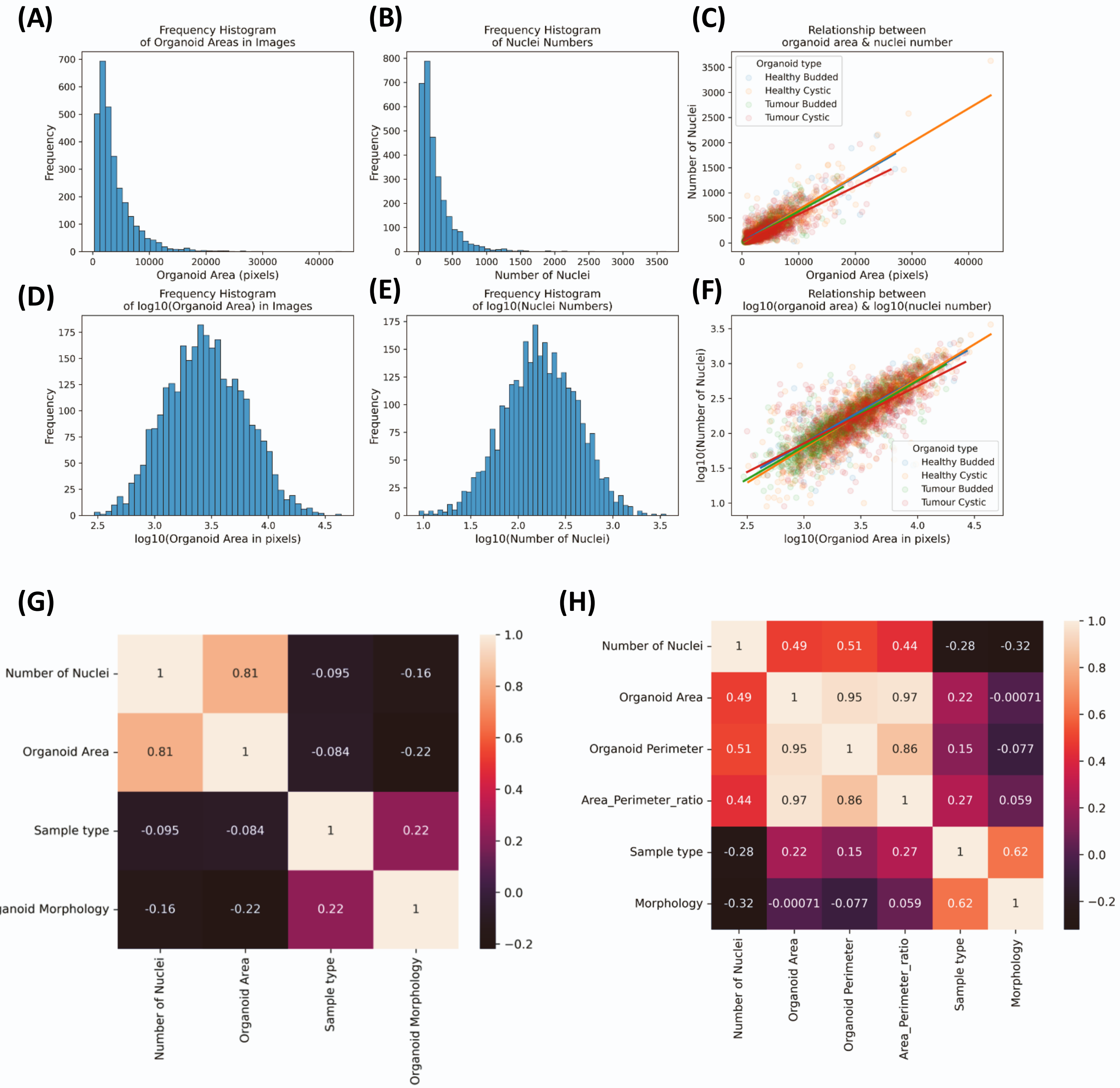

Supplementary Figure 3: Empirical linear model (eMLR) features. (A) Distribution of organoid area for organoids in the dataset. (B) Distribution of the number of nuclei in each organoid in the dataset. (C) Relationship between the area of an organoid and the number of nuclei in the same organoid, stratified by organoid type. (D) Distribution of log10-transformed organoid areas for organoids in the dataset. (E) Distribution of log10-transformed number of nuclei in each organoid in the dataset. (F) Relationship between log10-transformed organoid area and log10-transformed number of nuclei, stratified by organoid type. (G) Pairwise Pearson correlation matrix of numeric variables included in the empirical multiple linear regression model. (H) Pairwise Pearson correlation matrix of a test dataset which included organoid perimeter and the ratio of organoid area to organoid perimeter. The colour bars on the right of each matrix display the correlation r, with 1 being a very strong positive correlation and -1 being a very strong negative correlation. The correlation r is also displayed numerically at the centre of each square. All correlations displayed showed a statistical significance of less than 0.01. Sample type (healthy/tumour) is represented with 0 being healthy and 1 being tumour. Organoid morphology is represented with 0 being cystic (spherical) and 1 being budded (irregular). Related to Figure 2 and Figure 3.

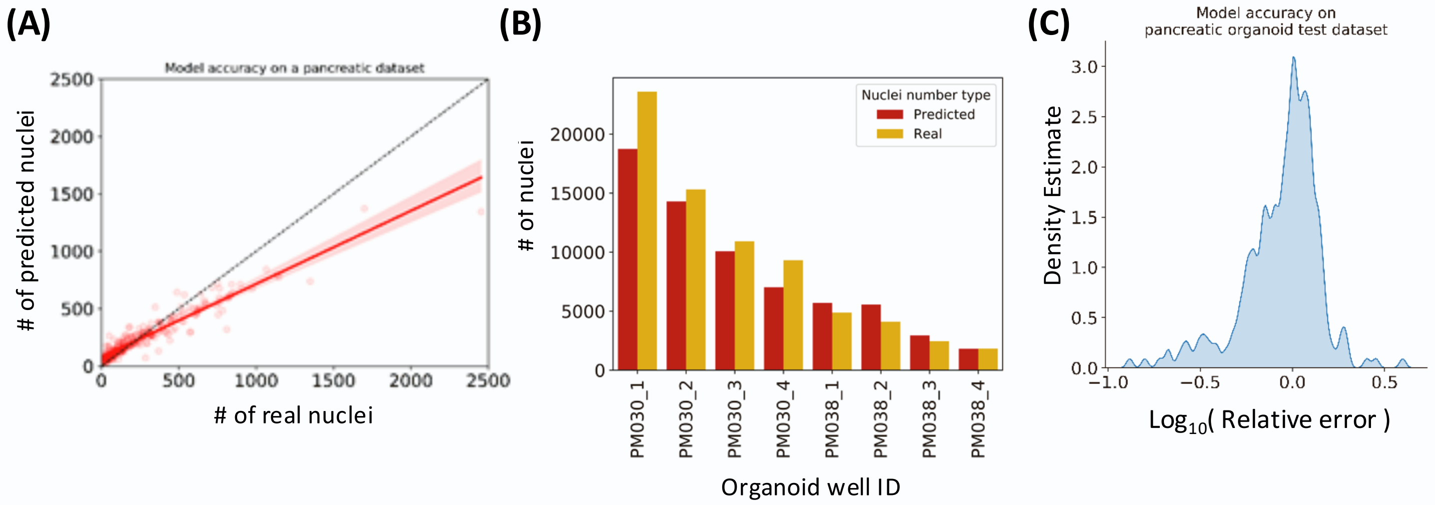

Supplementary Figure 4: Validating the ability of OSCAR to predict the number of cells within pancreatic organoids. (A) Comparison of the real number of nuclei in organoids versus the number predicted by OSCAR. (B) Comparison of the total number real cells in an organoid well to the predicted number of cells in a well. (C) Density distribution of the deviation in cell number predictions for pancreatic organoids in the dataset. Related to Figure 3.

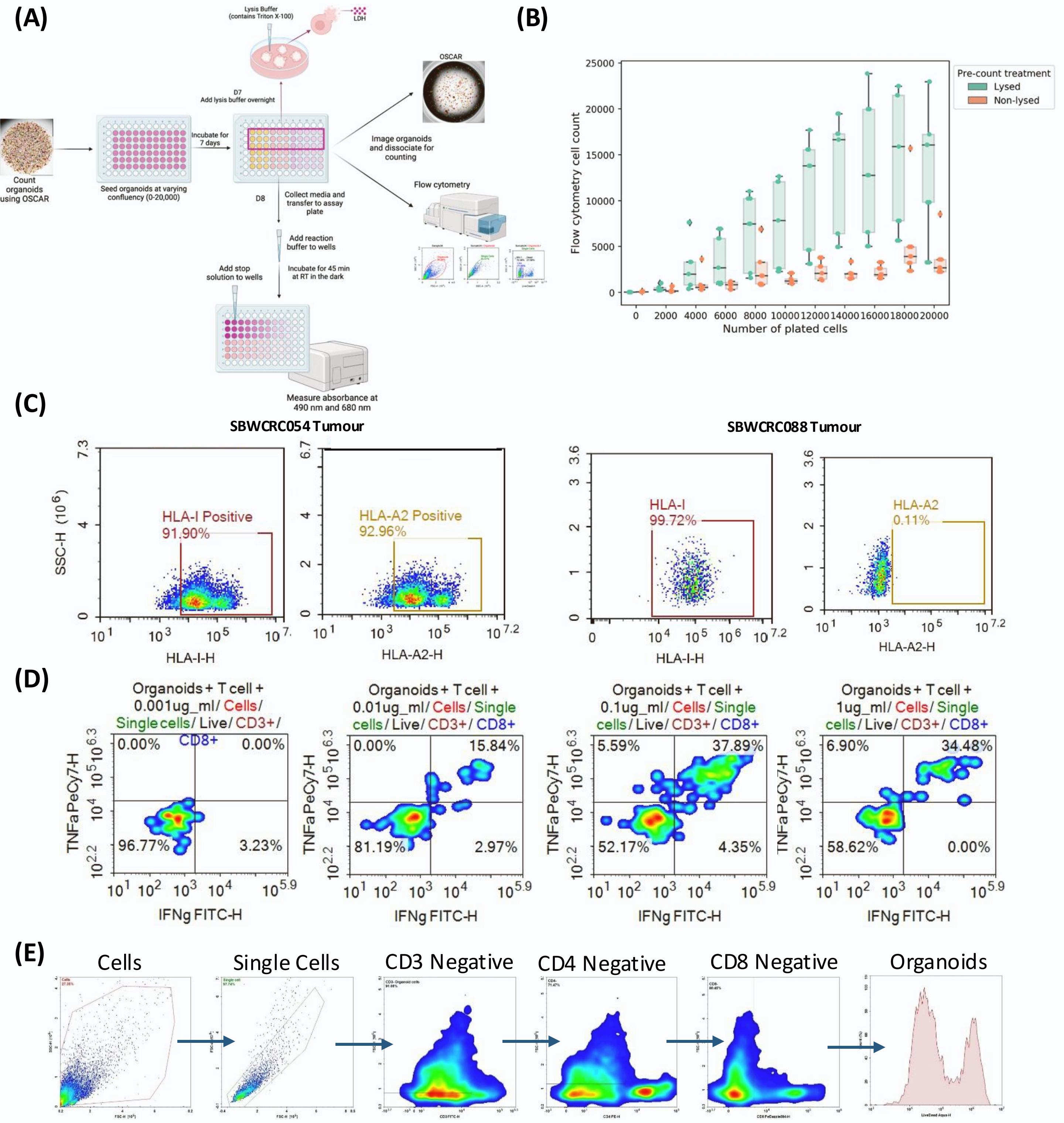

Supplementary Figure 5: Additional information about the flow cytometry of organoids: (A) A methodological summary of experiments comparing flow cytometry and OSCAR-generated organoid cell numbers to LDH measurements. Organoids were seeded at varying densities and cultured for 8 days. On day 7, a subset of organoids was imaged and counted using OSCAR, and lysis was performed to measure LDH. Supernatants were collected, and absorbance was measured at 490 nm and 680 nm. Non-lysed organoids were imaged, and all wells were dissociated for flow cytometry analysis of live/dead cells. LDH measurements were compared with OSCAR and flow cytometry cell counts to evaluate correlations. (B) Organoid cell counting using flow cytometry is prone to pre-processing-induced variation depending on the processing procedure. (C) Representative flow graphs to show organoids with and without HLA-I and HLA-A2 expression. (D) Organoids pulsed with HLA-I presented peptides can present peptide to CD8+ T cells to induce IFNγ and TNFα production. (E) Gating strategy to determine the proportion of live/dead organoid cells by gating out T cells and debris. Related to Figure 4 and 5.
